# Immunological Evaluation and Comparison of Different EV71 Vaccine Candidates

**DOI:** 10.1155/2012/831282

**Published:** 2012-09-12

**Authors:** Ai-Hsiang Chou, Chia-Chyi Liu, Jui-Yuan Chang, Shu-Pei Lien, Meng-Shin Guo, Hau-Pong Tasi, Kuang-Nan Hsiao, Shih-Jen Liu, Charles Sia, Suh-Chin Wu, Min-Shi Lee, Chia-Hsin Hsiao, Jen-Ren Wang, Yen-Hung Chow, Pele Chong

**Affiliations:** ^1^Vaccine R&D Center, National Institute of Infectious Diseases and Vaccinology, National Health Research Institutes, Zhunan Township, Miaoli County, Taiwan; ^2^Institute of Biotechnology, National Tsing Hua University, Hsinchu, Taiwan; ^3^Department of Medical Laboratory Science and Biotechnology, College of Medicine, National Cheng Kung University, Tainan, Taiwan; ^4^Graduate Institute of Immunology, China Medical University, Taichung, Taiwan

## Abstract

Enterovirus 71 (EV71) and coxsackievirus A16 (CVA16) are major causative agents of hand, foot, and mouth diseases (HFMDs), and EV71 is now recognized as an emerging neurotropic virus in Asia. Effective medications and/or prophylactic vaccines against HFMD are not available. The current results from mouse immunogenicity studies using in-house standardized RD cell virus neutralization assays indicate that (1) VP1 peptide (residues 211–225) formulated with Freund's adjuvant (CFA/IFA) elicited low virus neutralizing antibody response (1/32 titer); (2) recombinant virus-like particles produced from baculovirus formulated with CFA/IFA could elicit good virus neutralization titer (1/160); (3) individual recombinant EV71 antigens (VP1, VP2, and VP3) formulated with CFA/IFA, only VP1 elicited antibody response with 1/128 virus neutralization titer; and (4) the formalin-inactivated EV71 formulated in alum elicited antibodies that cross-neutralized different EV71 genotypes (1/640), but failed to neutralize CVA16. In contrast, rabbits antisera could cross-neutralize strongly against different genotypes of EV71 but weakly against CVA16, with average titers 1/6400 and 1/32, respectively. The VP1 amino acid sequence dissimilarity between CVA16 and EV71 could partially explain why mouse antibodies failed to cross-neutralize CVA16. Therefore, the best formulation for producing cost-effective HFMD vaccine is a combination of formalin-inactivated EV71 and CAV16 virions.

## 1. Introduction

Hand, foot and mouth diseases (HFMDs) caused by enteroviruses like Coxsackievirus A16 (CVA16) and Enterovirus 71 (EV71) infections have become serious public health problems in Southeast Asia [1–5]. Recent outbreaks of EV71 infections have led to fatalities and neurological complications in children, and the virus is now recognized as an important emerging infectious disease [3–5]. Anti-EV71 agents to control the disease, including vaccines, are presently being developed [3–6].

First isolated in 1969, EV71 is a nonenveloped RNA virus of the family Picornaviridae, 25–30 nm in diameter, that contains a single molecule of plus sense ssRNA (7.5–8.5 kb) and four structural proteins VP1, VP2, VP3, and VP4 [7–10]. Two structural proteins (VP1 and VP4) have been used for EV71 molecular genotyping and epidemiological monitoring. EV71 is currently classified into 3 genotypes A, B, and C. Genotypes B and C are further divided into B1-B5 and C1-C5 subgenotypes [9–12]. B5 isolates were recently identified in epidemics in Malaysia, Singapore, Taiwan, and Thailand. The virus strain that circulated in mainland China in the last few years was C4 [11, 12]. Based on the molecular epidemiological surveillance in Taiwan, CVA16 is the most common virus causing HFMD in children [12]. Therefore, an effective HFMD vaccine should elicit strong cross-neutralizing antibody responses against both EV71 and CVA16 in young children. 

The concern for potential virulent viruses reversed from attenuated vaccines [13, 14] has made chemically-inactivated virion, synthetic peptides, recombinant subunit, virus-like particles, and DNA vector-based vaccines as more favorable choices for EV71 vaccine development [3–6, 15–19]. Since there are no standardized antigens or immunological assays to reveal the potency of vaccine candidates, it is hard to compare the effective immune response of each method of EV71 vaccine development. In this study, using in-house standardized viral antigens and immunological assays, we report the immunogenicity results obtained from animals immunized with different vaccine candidates produced from various platform technologies. These EV71-based HFMD vaccine candidates include synthetic peptides containing virus neutralization epitopes, baculovirus expressed virus-like particles, recombinant EV71 subunit antigen produced from *E. coli*, and formalin-inactivated EV71 virus grown in Vero cell culture with and without animal serum containing media. Therefore, the results obtained from the current studies provide valuable information for future HFMD vaccine development.

## 2. Materials and Methods

### 2.1. Ethics Statement

All experiments were conducted in accordance with the guidelines of the Laboratory Animal Center of NHRI. The animal use protocols have been reviewed and approved by the NHRI Institutional Animal Care and Use Committee: NHRI-IACUC-098033-A.

### 2.2. Cells and Virus

African green monkey kidney (Vero) and rhabdomyosarcoma (RD) cell lines were purchased from the American Type Culture Collection (ATCC) (Manassas, VA, USA). Fetal bovine serum (FBS) was purchased from Moregate Biotech (Australia) and was certified by the manufacturer to be free of bovine spongiform encephalopathy (BSE). The E59 strain (genotype B4), the clinical isolate of the EV71 virus, was obtained from the Taiwan CDC. EV71 E59 virus stocks were collected from the supernatants of infected Vero cells at three days postinfection (DPI). Nucleotide sequences of VP1 reported in this study have been submitted to public domain (accession no. GQ150746.1), and the amino acid sequences of all four structural viral proteins reported in this study are available upon request. The EV71 subgenotype isolates used in this study are B4 (N0781-TW-01); B5 (N2838-TW-03); C4 (N3340-TW-02); CVA16 (N5079). These clinical virus isolates further underwent at least one round of plaque purification before growth in Vero and/or RD cells as virus seed stocks for virus neutralization assays.

### 2.3. Production of EV71 Virus in Vero Cell Grown in Serum-Containing or Serum-Free Medium

The production of EV71 virus was done using E59 vaccine strain in the presence of serum-containing medium (DMEM plus 5% fetal bovine serum) or VP-SFM medium (GIBCO, Carlsband, CA, USA) according to the procedures described in [18, 19]. Briefly, Vero cells (2 × 10^6^ cell per mL) grown in each roller bottle containing 200 mL of culture medium were infected with EV71/E59 at a multiplicity of infection (MOI) of 10^−5^. EV71 was collected from the culture supernatant of each bottle at five DPI. Cell debris were removed by filtration through a 0.65-*μ*m membrane (Sartorius Stedim Biotech, USA), and the crude virus bulk was concentrated 20- to 40-fold using a 100-kDa cut-off diafiltration membrane in a tangential flow filter (TFF) cassette (Sartorius Stedim Biotech). One liter of EV71 virus concentrate was purified using an AKTA Pilot liquid chromatography system (GE Healthcare, USA) equipped with Sepharose Fast Flow 6 gel. Fractions were collected and analyzed by immunoblotting, and virus infectivity was measured using the issue culture's infectious dose (TCID_50_) assay. Fractions containing the virus were pooled, further concentrated, and then inactivated with 0.02% formalin (v/v) at 37°C for 5 days. The vaccine bulk was obtained after sterile filtration using a 0.22 *μ*m filter, subjected to SDS-PAGE and western blot analyses, and stored at 4°C. The VP2 content was analyzed by Q-ELISA, and the total protein concentration of the vaccine bulk was also determined by the BCA protein assay. 

### 2.4. Determination of Viral Titer

Virus titers were determined using the median endpoint of the TCID_50_ as described previously by Liu et al. [20]. The TCID_50_ values were calculated using the Reed-Muench method [21].

### 2.5. Production of Recombinant EV71 Viral Antigens

The recombinant EV71 antigens (rVP1, rVP2, and rVP3) were expressed in *E. coli* BL21-DE3 and purified using Ni-NTA resin affinity chromatography (Qiagen, San Diego, CA, USA) as previously described by Liu et al. [20]. The purity of recombinant EV71 antigens was analyzed by SDS-PAGE and verified using anti-His tag antibody in the immunoblotting analysis. The concentration of each recombinant EV71 antigen was determined using a BCA protein assay, and the antigens were stored in a −20°C freezer. Different groups of BALB/c mice were immunized three times with 20 *μ*g of either individual recombinant antigen (rVP1, rVP2, and rVP3), or formulated with alum, or CFA/IFA adjuvant.

### 2.6. Production of EV71-Like Particles (EV71-VLPs) Using the Baculovirus Expression System

EV71-VLPs were produced using a recombinant baculovirus expression system using the P1 gene derived from the EV71 E59 virus isolate [19]. The EV71-VLPs were purified by ultracentrifugation using a CsCl density gradient. CsCl was removed by diafiltration using 300-kDa cut-off membranes. The EV71-VLPs were analyzed by SDS-PAGE and western blotting. Total protein concentrations were determined by a BCA protein assay. To test the potency of EV71-VLP, mice were immunized with 5 *μ*g of EV71 VLPs in the presence either of alum or CFA.

### 2.7. SDS-PAGE and Western Blot Analyses

SDS-PAGE and Western blot analyses of the purified viral antigens were performed according to the protocols reported previously by Liu et al. [22].

### 2.8. Peptide Synthesis

Peptide synthesis was performed by Kelowna International Scientific Inc. (Taipei Hsien, Taiwan) as previously described by Liu et al. [20]. To test whether these peptides could elicit immune responses, different groups of BALB/c mice were immunized three times with 50 *μ*g of either individual or mixed synthetic peptides formulated in either PBS, or alum, or complete Freund's adjuvant (CFA)/incomplete Freund's adjuvant (IFA). The reactivity of the antibody (total IgG titer) to synthetic peptide was analyzed by ELISA according to the protocol previously reported by Panezutti et al. [23].

### 2.9. Animal Immunogenicity Studies

Immunogenicity studies were conducted according to the protocols reported previously by Liu et al. [19]. Briefly, different concentrations of the EV71 vaccine candidates (synthetic peptides, individual recombinant viral protein, VLP, formalin-inactivated virion) were mixed with aluminum phosphate at room temperature for three hours before immunization. Groups of 3 to 6 female BALB/c mice (6–8 weeks old) were immunized intramuscularly (i.m.) with 0.2 mL of different dosages of EV71 immunogens. The animals were boosted with the same dose at two-week intervals after priming. Immunized mice were bled one week after the final boost, and the serum was collected and stored at −80°C. In parallel, rabbits were immunized i.m. with 0.5 to 20 *μ*g of EV71 protein formulated with 1.5 mg of alum per dose. Sera were collected two weeks after each immunization and used for immunological analysis. The specificity and anti-EV71 titer of the antisera were tested by western blotting and TCID_50_-based virus neutralization assay, respectively.

### 2.10. Virus Neutralization Assay

Virus neutralization titer of each serum sample was determined using RD cell and in-house standardized TCID_50_ assay according to the protocols reported previously by Liu et al. [19]. The virus isolates underwent at least one round of plaque purification in Vero cell culture before growth in RD cells as the standardized virus seed stocks for virus neutralization assay.

### 2.11. Recombinant Viral Protein-Specific Enzyme-Linked Immunosorbent Assay (rVP-ELISA)

The rVP-ELISA was performed according to the protocol reported previously by Liu et al. [20]. 

## 3. Results 

### 3.1. Mouse Immune Response to EV71 Synthetic Peptides

Our recent studies have identified two linear immunodominant neutralization epitopes VP1-43 and VP2-28 that, respectively, correspond to residues 211–225 of VP1 and residues 136–150 of VP2 [20]. Although both synthetic peptides in the presence of CFA/IFA adjuvant induced antibody reactions in peptide-ELISA and western blot analysis (Table 1), VP2-28 was found to be less immunogenic. VP1-43 alone or mixed with VP2-28 in the presence of CFA elicited antibody response with low virus neutralization titer (1/32) against EV71 E59 isolate (B4 subgenotype), while synthetic peptide VP2 did not (Table 1). Interestingly, the current results were consistent with previous reports by Foo et al. [24, 25] that synthetic peptide (SP70) containing residues 211–215 of VP1 elicited virus-neutralizing antibody response (1/32 titer) and protected newborn mice against lethal EV71 challenges by passive immunization. Although there was 60% homology between EV71 and CVA16 at the VP1-43 peptide sequence [20], EV71 VP1-43 specific antibodies failed to neutralize CVA16 at 1/8 serum dilution. C56BL/6 mouse immunogenicity studies were also performed and the results were very similar to those obtained from BALB/c mice. So far there is no evidence indicating that antibodies generated from EV71 peptides can neutralize CVA16.

### 3.2. Immunogenicity Study of Recombinant Viral Structural Proteins

Based on the structural organization of the polio virus, VP1, VP2 and VP3 are exposed on the viral surface, while VP4 is buried inside the capsid. Thus, VP1, VP2, and VP3 antigens are the targets for EV71 subunit vaccine development. To compare their murine immune responses, rVP1, rVP2, and rVP3 were produced and purified using the protocol previously described [20]. Twenty micrograms of individual recombinant EV71 antigens formulated either with alum or alone could induce mouse antibody recognition of its respective protein in the western blot analysis (Table 1). Mice immunized with rVP1 alone or formulated in alum generated IgG antibodies, reacting with VP1-43 peptide in the peptide-ELISA, but these antibodies did not neutralize EV71 virus *in vitro* (Table 1). In contrast, using CFA/IFA as adjuvant recombinant VP1 elicited antibody responses that have 1/128 virus neutralization titer against EV71 B4 subgenotype (Table 1). Mice immunized with either rVP2 or rVP3 formulated with CFA/IFA adjuvant produced strong antibody responses against itself, but surprisingly these antibodies had very weak neutralization titers (1/8) against EV71 (Table 1). 

A recent report by Liu et al. [26] indicated that mice immunized with 100 *μ*g of *E. coli* expressed recombinant antigen sequences comprising *ca.* 100 amino acids from VP2 and VP3 (P140-249, P230-323, P324-443, and P444-565) in the presence of CFA/IFA adjuvant also produced weak virus-neutralizing antibody responses against EV71. The titers were found to range from 1/32 to 1/64. Again, these EV71 viral antigen-specific antisera failed to neutralize CVA16 at 1/8 serum dilution. These results suggest that there were no CVA16 cross-neutralizing antibodies elicited from recombinant antigens.

### 3.3. Mouse Immunogenicity Studies of EV71-VLP

Since a handful of prophylactic VLP-based vaccines against hepatitis B virus and human papillomavirus are currently commercially available, many VLP-based vaccine candidates against different diseases are in clinical trials or in preclinical evaluations [27]. To this end, EV71 VLPs were produced from recombinant baculovirus and purified as previously reported [19]. Mice immunized with 5 *μ*g of EV71 VLPs in the presence either of alum or CFA produced antibodies with virus neutralization titers of 1/128 and 1/160, respectively. Interestingly, EV71 VLP alone also elicited a virus-neutralizing antibody response with a 1/64 titer. The current results are consistent with previous reports by Chung et al. [8] that 10 *μ*g of EV71 VLPs formulated with CFA could elicit virus neutralizing antibody responses with a titer of 1/512 in adult mice and protect newborn mice against lethal EV71 challenges by passive immunization. Again, these EV71 VLP-specific antisera were found to fail to neutralize CVA16 at 1/8 serum dilution. C56BL/6 mouse immunogenicity studies were also performed and the results were very similar to those obtained from BALB/c mice. These results suggest that while EV71 VLPs may fully mimic the structural organization of EV71, the structural similarity does not elicit mouse antibodies that are capable of cross-neutralizing CVA16.

### 3.4. Mouse Immunogenicity Studies of Formalin-Inactivated EV71 Virion

**Figure 1:**
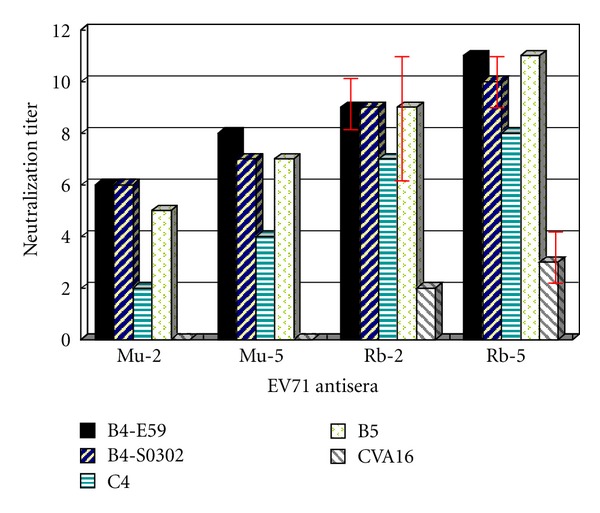
The average crossneutralization antibody titers (log 2 × 10) elicited by different amounts of inactivated EV71 virions in mice and rabbits. Mu-2, Mu-5, Rb-2, and Rb-5 are antisera obtained from mice (pooled sera from 3 immunized mice) and rabbits (2 rabbits) immunized 3 times with 2 or 5 *μ*g of inactivated EV71 vaccine candidate formulated with alum as adjuvant. The name code of each virus isolate is described in Materials and Methods.

Similar to our previous reports [18, 20, 22], 2 *μ*g of formalin-inactivated EV71 virion alone or formulated in alum elicited antibody responses that (1) recognized viral structural proteins in western blot analysis, (2) reacted specifically with VP1-43 neutralization epitope, and (3) neutralized EV71 B4 genotype with average titers ranging from 1/64 (alone) to 1/640 (alum), but (4) failed to neutralize CVA16 (Table 1). Antisera generated from mice immunized with 5 *μ*g of the formalin-inactivated EV71 virion formulated in alum strongly cross-neutralized different genotypes of EV71 virus (Figure 1) with the highest titer at 1/2560, but was still not able to neutralize CVA16 (Table 1). There were surprising results from the western blot analyses: mouse antisera did not recognize any CVA16 viral protein bands in the western blot analysis and a CVA16 peptide corresponding to residues 210–225 in the peptide-ELISA study (no reactivity at 1/200 dilution). In our previous study [20], we demonstrated that murine immunodominant neutralizing antibodies specifically reacted with the neutralization epitope (residues 210–225) and its biological function was totally inhibited by the synthetic peptide VP1-43. Since the amino acid sequence obtained from EV71 isolates were highly conserved (Table 3), it was not surprising that the antisera could cross-neutralize different EV71 genotypes (Figure 1). In contrast, as shown in Table 3 the amino acid sequence dissimilarity between CVA16 and EV71 could explain the current observation that mouse antibodies failed to cross-neutralize CVA16, despite having similar structural organization, but different conformation.

### 3.5. Rabbit Immunogenicity Studies of Different EV71 Vaccine Candidates

Similar to our previous reports [18, 19], 2 *μ*g of the formalin-inactivated EV71 virion alone or formulated in alum could elicit rabbit antibody responses that recognized EV71 viral structural proteins in the western blot analyses and reacted specifically with VP2-28 neutralization epitope in the peptide-ELISA, but reacted poorly with VP1-43 peptide as shown in Table 2. The rabbit antisera also strongly cross-neutralized different EV71 genotypes, with average titers ranging from 1/1280 (against C4 subgenotype) to 1/5120 (against B4 and B5 subgenotypes). However, the rabbit antisera only weakly neutralized CVA16 with a titer of 1/32 (Table 2). Although antisera generated from rabbits immunized with 5 *μ*g of the formalin-inactivated EV71 candidate resulted in enhanced cross-neutralization titers (4 fold) against different EV71 genotypes (Figure 1), these antisera could not significantly increase the neutralization titer against CVA16 (1/64). Unlike mouse antisera, rabbit antisera recognized CVA16 viral protein bands in western blot analyses (VP0, VP1, and VP3). These results indicate that rabbit antisera unlike those obtained from mice recognized more than one linear immunodominant epitope. Our previous reports [19, 20] indicated that rabbit antisera reacted specifically with a linear immunodominant epitope VP2-28 that could not effectively block viral neutralization activities of rabbit antisera. Therefore, rabbit antisera containing diverse neutralizing antibodies most likely recognized conformational epitopes in EV71 virion. Since there are 60 to 80% amino acid sequence similarity between EV71 and CVA16, it is not surprising that the antisera could cross-react with CVA16 in western blot analyses. The difference in the viral structural protein sequences may also explain why rabbit antibodies did not effectively cross-neutralize CVA16, despite having similar structural organization, but different conformation from EV71.

## 4. Discussion

EV71 and CVA16 are two major causative agents of HFMD, and effective medications and prophylactic vaccines against HFMD are not available.EV71 vaccine candidates based on chemically-inactivated virion, synthetic peptides, recombinant subunit, virus-like particles and DNA are being developed and reported in literature [3–6, 15–19]. Since there are no standardized antigens or immuno assays to reveal the potency of vaccine candidates, it was difficult to compare which method of EV71 vaccine development elicited the most effective immune response. In this study, using in-house standardized viral antigens and immunological assays (RD cell microneutralization assay) we compared the immunogenicity results obtained from animals immunized with different vaccine candidates produced from various platform technologies. Based on our results, we can rank the candidates in the following order by potency and efficacy: formalin-inactivated virion > recombinant virus-like particles > recombinant VP1 > synthetic peptide containing EV71 neutralization epitope (VP1-43). Fully synthetic peptides containing neutralization epitopes could (a) provide well-defined and cost-effective immunogens and safety advantages; (b) reduce any potential unwanted immune responses, such as antibody-dependent enhancement (ADE), that were reported in two recent papers [28, 29]; and (c) promote EV71 vaccine usage in developing countries due to the cost of products. Like other reports [24, 25], only CFA/IFA adjuvant-formulated synthetic peptides containing VP1-43 epitope could elicit weak (1/32 titer) and strain-specific viral neutralizing antibody responses in mice. In contrast, VP1-43 formulated in CFA/IFA adjuvant was not immunogenic in rabbit immunogenicity studies (data not shown). Low neutralization titer and the need for CFA/IFA adjuvant make synthetic peptide-based EV71 vaccine less attractive.

It is well accepted that recombinant subunit vaccines are safe and cost-effective, but require strong adjuvants. Several studies have reported that recombinant VP1 (rVP1) antigen produced and purified from different expression systems could elicit strong virus neutralizing antibody responses and protect newborn mice against lethal EV71 challenge [3–5]. However, there are not many studies which report the immunogenicity of recombinant VP2 and VP3 antigens. In current murine immunogenicity studies we found recombinants VP1, VP2, and VP3 to be immunogenic and are capable of eliciting antibodies that recognize their respective viral proteins. Only recombinant VP1 was capable of eliciting an antibody response with 1/128 virus neutralization titer against EV71 in the presence of CFA/IFA as an adjuvant, but these antibodies failed to neutralize CVA16. The amino acid sequence dissimilarity between CVA16 and EV71 (Table 3) could explain the result that mouse antibodies failed to cross-neutralize CVA16. These results suggest an HFMD vaccine based on recombinant VP1 would not be viable or attractive since the antisera cannot neutralize CVA16.

Recombinant virus-like particles produced from baculovirus or yeast expression systems have structural organization mimicking the conformation of authentic native viruses, but lack the viral genome to potentially provide safer vaccines. In addition, prophylactic VLP-based vaccines against hepatitis B virus and human papillomavirus are currently commercially available. Our results are consistent with previous reports by Chung et al. [8] that 10 *μ*g of EV71 VLPs could elicit virus-neutralizing antibody responses with a titer of 1/512 in adult mice and protect newborn mice against lethal EV71 challenges by passive immunization. Although these EV71 VLP-specific antisera were capable of cross-neutralizing other EV71 genotypes as determined by peptide-ELISA and Western blot analysis and have higher EV71 neutralization titers than those obtained from synthetic peptide or recombinant VP1, it still failed to neutralize CVA16 at 1/8 serum dilution. These results suggest that EV71 VLP may have structural similarity and fully mimic the EV71 structural organization, but could not elicit mouse antibodies that can cross-neutralize CVA16. Again, the amino acid sequence dissimilarity in CVA16 could explain this observation since mouse antibodies recognize the single immunodominant epitope VP1-43. In fact CVA16 has several substitutions in this location, in particular those lysine residues are found to be critical for neutralizing antibody binding [20].

The concern over potential virulent viruses reversed from attenuated vaccines [13, 14] has made chemically inactivated virion-based vaccines a more favorable choice for EV71 vaccine development. Currently there are several inactivated EV71 vaccine candidates in clinical trials [6]. In our previous reports [18, 20, 22], we evaluated the potency of inactivated EV71 virion produced from either serum-containing or serum-free medium in different animal immunogenicity studies. It was of interest to determine whether vaccine candidates containing formalin-inactivated EV71 virion could induce superior immune responses compared to other vaccine candidates mentioned above. The formalin-inactivated EV71 virion elicited antibody responses that cross-neutralized different EV71 genotypes with neutralization titers ranging from 1/64 to 1/1280, but failed to neutralize CVA16. In contrast, antisera generated from rabbits immunized with the same amount (2 *μ*g) of inactivated EV71 virion strongly cross-neutralized different EV71 genotypes and weakly cross-neutralized against CVA16, with average titers of 1/6400 and 1/32, respectively. Our current results are consistent with results reported by both Bek et al. and Dong et al. [30, 31] which produced formalin-inactivated EV71 vaccine candidates based on the C4 genotype virus. These studies also found these vaccine candidates to be highly immunogenic and could elicit cross-genotype neutralizing antibody responses in mice and nonhuman primate models. Taking these results together, we can conclude that the potent and important cross-genotype neutralization epitopes of EV71 virus were conformational and may not be mimicked by synthetic peptides or recombinant subunit antigens. Furthermore, the current results suggest that common neutralization epitopes (most likely conformational) exist in EV71 virus and contribute to eliciting strong antibody responses that are capable of cross-neutralizing different EV71 genotypes. In addition, the amino acid sequence dissimilarity between EV71 and CVA16 can partially explain the observation that mouse antibodies failed to cross-neutralize CVA16 (which may have similar structural organization like EV71). It is of interest to know whether inactivated CVA16 virions could elicit cross-neutralizing antibodies against different EV71 genotypes. These studies are currently ongoing. Therefore, based on current information the best formulation for producing a stable and cost-effective of HFMD vaccine is a combination of formalin-inactivated EV71 and CAV16 virions. 

## Figures and Tables

**Table 1:** Summary of mouse immunogenicity studies with different EV71 vaccine candidates.

EV71 vaccine candidates	Adjuvant formulated	Total IgG titers		Western blot			Virus neutralization titer	
		VP1-43	VP2-28	rVP1	rVP2	rVP3	EV71 (B4 E59)	CVA16 (N5079)
VP1-43	Alum	1/3200		++			<1/8	<1/8
VP1-43	CFA/IFA	1/6400		+++			1/32	<1/8
VP2-28	Alum		1/200		−		<1/8	<1/8
VP2-28	CFA/IFA		1/1600		+		<1/8	<1/8
VP1-43/VP2-28	CFA/IFA	1/6400	1/800	+++	+		1/32	<1/8

rVP1	Alum	1/6400		+++			<1/8	<1/8
rVP1	CFA/IFA	1/12800		+++			1/128	<1/8
rVP2	Alum		<1/100		+++		<1/8	<1/8
rVP2	CFA/IFA		1/200		+++		1/8	<1/8
rVP3	Alum					++	<1/8	<1/8
rVP3	CFA/IFA					+++	1/8	<1/8

EV71-VLP	PBS	1/1600	<1/100	++	++	++	1/64	<1/8
EV71-VLP	Alum	1/6400	<1/100	++	++	++	1/128	<1/8
EV71-VLP	CFA/IFA	1/12800	<1/100	+++	+++	++	1/160	<1/8

2 *μ*g of inactivated								
EV71 virion	PBS	1/6400	<1/100	+++	+++	++	1/64	<1/8
EV71 virion	Alum	1/12800	<1/100	+++	+++	++	1/640	<1/8

5 *μ*g of inactivated								
EV71 virion	Alum	1/12800	<1/100	+++	+++	+++	1/2560	<1/8

Three to six mice per group were immunized 3 times with EV71 vaccine candidates formulated with alum or CFA/IFA. Fifty micrograms of synthetic peptides, 20 *μ*g of recombinant EV71 viral structural proteins, and 5 *μ*g of recombinant virus-like particles (EV71-VLP) were used in the mouse immunogenicity studies. The protocols for IgG titer determination, western blot analysis, and virus neutralization assay are described in Section 2.

**Table 2:** Summary of rabbit immunogenicity studies with different EV71 vaccine candidates.

EV71 vaccine candidates	Adjuvant formulated	Total IgG titers		Western blot			Virus neutralization titer	
		VP1-43	VP2-28	rVP1	rVP2	rVP3	EV71 (B4 E59)	CVA16 (N5079)
2 *μ*g of inactivated								
EV71 virion	PBS	1/200	1/800	+++	+++	++	1/128	<1/8
EV71 virion	Alum	1/200	1/6400	+++	+++	++	1/6400	1/32

5 *μ*g of inactivated								
EV71 virion	Alum	1/200	1/6400	+++	+++	+++	1/12800	1/64

Two to three rabbits per group were immunized 3 times with EV71 vaccine candidates alone or formulated with alum. The protocols for IgG titer determination, western blot analysis, and virus neutralization assay are described in Section 2.

**Table 3:** Alignment of VP1 amino acid (200–225) sequences from different EV71 subgenotypes and CVA16.

Strains	EV71 subgenotype or CVA16	Sequences
BrCr	A	QWFYDGYPTFGEHKQEKDLEYGAC
238/TW66	B1	QWFYDGYPTFGEHKQEKDLEYGAC
7423/CT/87	B2	QWFYDGYPTFGEHKQEKDLEYGAC
EV71/SAR/SHA66	B3	QWFYDGYPTFGEHKQEKDLEYGAC
EV71/9/97/SHA89	B4	QWFYDGYPTFGEHKQEKDLEYGAC
N2838-TW-03	B5	QWFYDGYPTFGEHKQEKDLEYGAC
1M/AUS/12/00	C1	QWFYDGYPTFGEHKQEKDLEYGAC
TW/2086/98	C2	QWFYDGYPTFGEHKQEKDLEYGAC
Kor/EV71/10	C3	QWFYDGYPTFGEHKQEKDLEYGAC
N3340-TW-02	C4	QWFYDGYPTFGEHKQEKDLEYGAC
EV71 E59	B4	QWFYDGYPTFGEHKQEKDLEYGAC
Tainan/5079/98	CVA16	QWFYDGYPTFGEH**L**Q**AN**DL**D**YG**Q**C
